# Carbon monoxide and a change of heart

**DOI:** 10.1016/j.redox.2021.102183

**Published:** 2021-11-08

**Authors:** Louis M. Chu, Shazhad Shaefi, James D. Byrne, Rodrigo W. Alves de Souza, Leo E. Otterbein

**Affiliations:** a Harvard Medical School, Departments of Surgery, Critical Care and Pain Medicine, Beth Israel Deaconess Medical Center, Boston, MA, 02215, USA; b Departments of Anesthesia, Critical Care and Pain Medicine, Beth Israel Deaconess Medical Center, Boston, MA, 02215, USA; c University of Iowa, Iowa City, IA, 52242, USA

## Abstract

The relationship between carbon monoxide and the heart has been extensively studied in both clinical and preclinical settings. The Food and Drug Administration (FDA) is keenly focused on the ill effects of carbon monoxide on the heart when presented with proposals for clinical trials to evaluate efficacy of this gasotransmitter in a various disease settings. This review provides an overview of the rationale that examines the actions of the FDA when considering clinical testing of CO, and contrast that with the continued accumulation of data that clearly show not only that CO can be used safely, but is potently cardioprotective in clinically relevant small and large animal models. Data emerging from Phase I and Phase II clinical trials argues against CO being dangerous to the heart and thus it needs to be redefined and evaluated as any other substance being proposed for use in humans. More than twenty years ago, the belief that CO could be used as a salutary molecule was ridiculed by experts in physiology and medicine. Like all agents designed for use in humans, careful pharmacology and safety are paramount, but continuing to hinder progress based on long-standing dogma in the absence of data is improper. Now, CO is being tested in multiple clinical trials using innovative delivery methods and has proven to be safe. The hope, based on compelling preclinical data, is that it will continue to be evaluated and ultimately approved as an effective therapeutic.

## 1 A history of CO and cardiotoxicity

Carbon monoxide (CO) is readily recognized for its pollutant and toxic effects, and is the most common cause of death by poisoning in the United States, where it is responsible for over 400 deaths, 2,000 hospitalizations, and 21,000 emergency department visits annually [1]. It is a colorless, odorless gas that binds to hemoglobin with a 200-fold greater affinity than oxygen (O_2_), blocking O_2_ binding and decreasing O_2_ transport capacity [2]. CO binding to one heme group also increases the affinity of the other heme groups to O_2_, resulting in a left shift in the hemoglobin-O2 dissociation curve thereby reducing the release of O_2_ in tissues [3]. In mitochondria, CO inhibits cytochrome *c* oxidase, impairing ATP synthesis and increasing production of reactive oxygen species.

The average concentration of CO in homes is <2 parts per million (ppm) [[4], [5], [6], [7], [8]]. Near gas stoves the CO concentration rises to 15 ppm, while wood fires generate about 5000 ppm and car exhaust contains about 7000 ppm [[9], [10], [11], [12], [13]]. The amount of carboxyhemoglobin generated by CO exposure is dependent on a myriad of variables, most notably the CO concentration in the inspired air, the duration of exposure and alveolar ventilation. Other factors contributing significantly include diffusion capacity, hemoglobin mass and blood volume. Normal levels of circulating carboxyhemoglobin are typically lower than 2–3%, but in smokers can be as high as 5–13% [14]. Those with underlying cardiac or respiratory dysfunction may be particularly predisposed to the toxic effects of CO as they may already be sensitive to cardiac ischemia or have impaired pulmonary gas exchange.

**Fig. 1:**
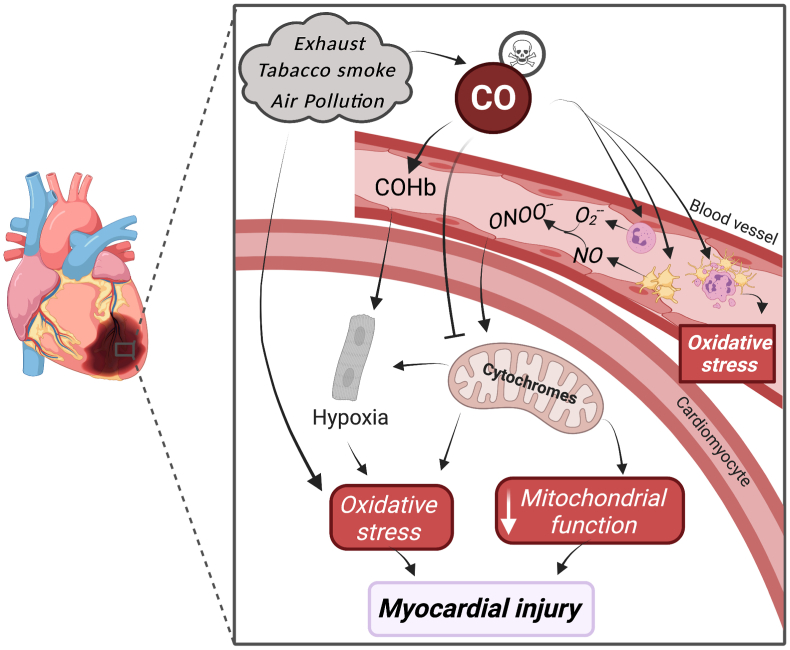
**Pathophysiology of carbon monoxide poisoning in the heart.** CO diffuses rapidly into the bloodstream as a component of inhaled pollutants. In addition to binding to hemoglobin, CO modulates platelet function to increase nitric oxide (NO) production. NO reacts with oxygen free radicals (O_2_^−^) to produce peroxynitrite (ONOO^−^), inhibiting mitochondrial function and further activating platelets and neutrophils. CO can induce platelet-neutrophil aggregation, neutrophil degranulation, release of myeloperoxidase, proteases, and additional reactive oxygen species, that contribute to tissue injury, release DAMPs, and increase cell death. Ultimately CO exposure is thought to limit O_2_ availability to highly metabolic cardiac myocytes that in situations where the heart is compromised (e.g. angina, ischemia/reperfusion injury) can exacerbate symptoms and contribute to further ischemic tissue damage. Importantly, exhaust (fossil fuel combustion) contains hundreds of pro-inflammatory and hazardous compounds including hydrocarbons, carbon particulate, sulfur dioxide, and nitrogen dioxide all of which can exacerbate cardiac tissue survival and function.

Myocardial tissue is highly sensitive to oxygen deprivation; thus when CO displaces O_2_ to form COHb, it can be uniquely detrimental to the heart especially in those with diseases that compromise heart function such as coronary artery disease, cardiomyopathy, or myocardial infarction [[15], [16], [17]]. The belief when considering exposure to CO is that it is exceptionally hazardous to cardiac tissue. (Fig. 1). Cardiovascular manifestations of CO toxicity as reported in the literature include arrhythmias, pulmonary edema, heart failure, and MI, but myocardial dysfunction usually dissipates within 24 h of exposure [18,19]. Chronic exposure to low level CO, as in environmental pollution, results in cardiac hypertrophy, elevated heart rate, and impaired contractility, perhaps as a result of increased oxidative stress (Fig. 1) [20]. In addition to hypoxic effects on myocardium, CO also impairs mitochondrial ATP formation, forcing myocytes to switch to anaerobic metabolism, worsening lactic acidosis and apoptosis. What is typically less mentioned are the large number of agents in exhaust and cigarette smoke that also contribute to heart disease and would include substances such as sulfur dioxide, hydrocarbons and even particulate matter [[21], [22], [23]]. CO has also been shown to increase endothelial cell apoptosis [24] and oxidative stress [25], resulting in coronary vasoconstriction and worsening myocardial perfusion. In addition, CO increases calcium sensitivity and diastolic retention in myocytes, predisposing the heart to arrhythmias [20]. The dichotomy between the beneficial and detrimental effects of CO in the heart is clear. Thus, caution must dictate the path forward, as CO proceeds through clinical testing and as is done with all drug candidates.

To date, very little preclinical data using low, well-tolerated doses of CO suggests adverse effects of CO on the heart. In fact, stressing the heart increases the amount of HO-1 and thus endogenous CO in myocytes, which should support the notion that CO is in all likelihood not cardiotoxic. Had medical researchers abandoned NO simply because it was a component of exhaust, thousands of neonates would suffer unnecessarily from pulmonary hypertension and perhaps millions of adults from unstable angina.

## 2 Therapeutic carbon monoxide in myocardial ischemia – prelude to clinical trials

**Fig. 2:**
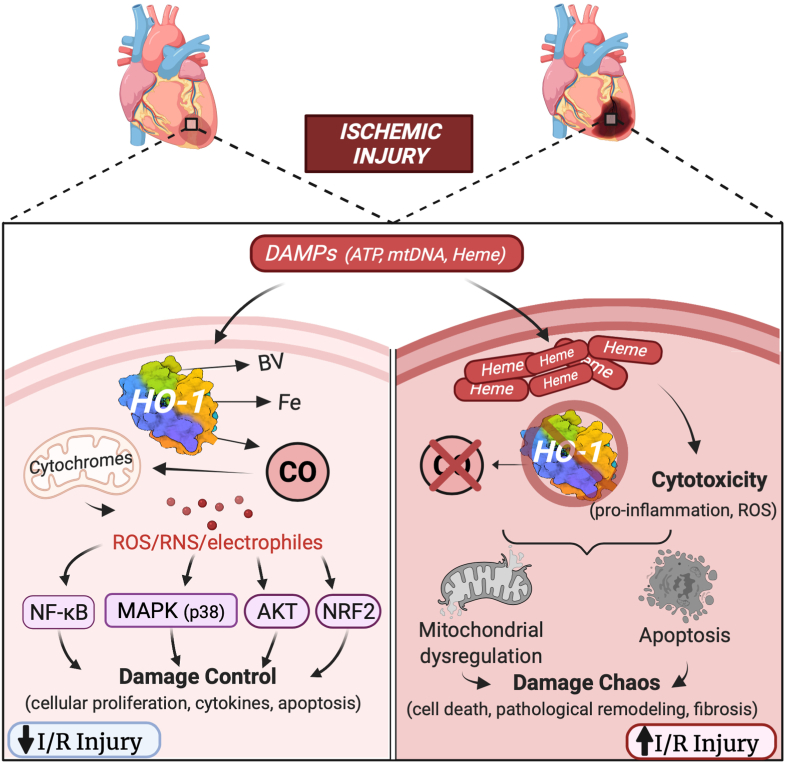
Protective role of CO in the heart and vasculature. Tissue injury leads to the sudden release of cellular contents, including high levels of heme. These cellular danger-associated molecular patterns (DAMPs) have been shown to induce heme oxygenase-1 (HO-1) and the subsequent generation of endogenous CO. When induced, HO-1 is highly cardioprotective and this can be mimicked using exogenous CO. Because low concentrations of CO partially and transiently bind to cytochrome *c* oxidase, low levels of ROS are generated that activate signaling complexes that include nuclear factor erythroid 2 like 2 (NFE2L2; NRF2), HIF-1α, and NF-κB/IκB. Moreover, CO upregulates expression of peroxisome proliferator-activated receptor-gamma coactivator-1α (PGC-1α) and the nuclear respiratory factor-1 (NRF1) and NRF2, which further regulate expression of nuclear encoding mitochondrial proteins. These factors are all thought to be involved in CO-induces mitochondrial biogenesis that results in tissue preservation and regeneration. In the vasculature, including in the heart, the effects of CO on vasodilatation are mediated in part by the soluble guanylate cyclase (sGC)/cyclic 3′:5′-guanosine monophosphate (cGMP) and protein kinase-G (PKG). Further, CO has been shown to inhibit synthesis of the potent vasoconstrictor endothelin-1, prevent platelet aggregation, and inhibit L-type Ca^2+^ channels resulting in cytoprotection of cardiomyocytes.

**Fig. 3:**
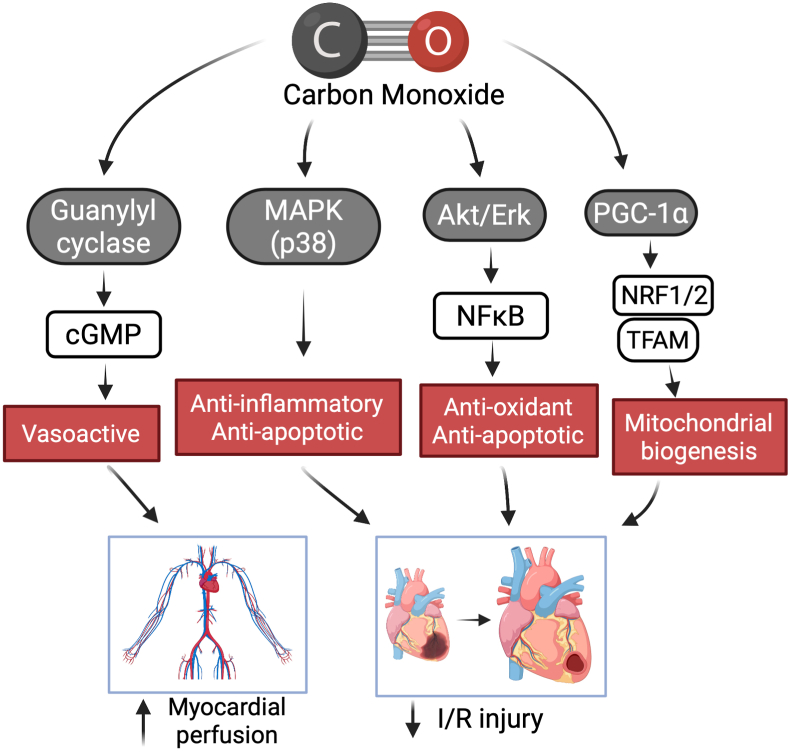
**Proposed Mechanisms of action for CO in the heart and vasculature**. Both heme-dependent (e.g. guanyl cyclase) and heme-independent pathways (e.g. MAP kinases, Akt/Erk, and *nuclear factor erythroid 2 like 2, NRF2*) in regulation of perfusion, inflammation, cell death, mitochondrial biogenesis and oxidative stress. I/R: ischemia/reperfusion.

Nonetheless, many studies over the past 20 years have incrementally investigated the potential therapeutic applications of low dose, exogenously administered CO in myocardium. The history of CO investigation can be traced back to as early as 1949, where several groups showed that CO is also produced endogenously, particularly in conditions of increased red blood cell breakdown [26,27]. In 1969, Tenhunen first described the function of the heme oxygenases (HOs), which catalyze the breakdown of heme into CO, iron, and biliverdin [28]. Specifically in myocardium, the upregulation of inducible HO-1 in response to ischemia-reperfusion injury has been shown to be an important mediator of cardioprotection and may operate not only by generating CO, but metabolizing cytotoxic heme [29,30]. Absence of HO-1 dramatically increases susceptibility to ischemic injury [31] (Fig. 2). HO-1 acts, in part by generating bioactive products including CO that maintains damage control with tissue recovery and repair, but when HO-1 is absent the injury is exaggerated and chaos results (Fig. 2). Administration of exogenous CO can mimic HO-1 induction and decrease infarct size, reduce apoptosis, and increase inotropy in the rat heart [[32], [33], [34]]. Though the specific mechanisms behind these effects remain ill-defined, Zuckerbraun et al. demonstrated in 2007 that the anti-inflammatory effects of CO disappear in respiration-deficient cells lacking mitochondria [35]. Further mechanistic studies by Piantadosi et al. showed that CO functions in part through an Akt-GSK-3β-nrf2 pathway that results in mitochondrial biogenesis. Further that HO-1/CO effects on mitochondria are essential in maturation of stem cells into cardiac myocytes in the heart [36,37]. Fig. 3 depicts some of the known signaling pathways in the heart and blood vessels involved in abrogating tissue injury. In fact, improving mitochondrial density, impacts capillary density in skeletal muscle [38]. Thus myocardial mitochondrial function is likely at the crux of CO-induced cardioprotection and linked to downstream signaling, gene regulation and cell survival.

Clinical trials investigating the cardioprotective properties of CO have been limited by its potential toxicity, poor targetability, and inconsistent delivery in non-intubated patients. Innovative new delivery systems for inhaled, topical, and parenteral dosing of CO have allowed for safer and more specific delivery of the therapeutic gas to the cardiovascular system. This review will discuss some of the strategies that may make clinical trials of therapeutic CO safer and more feasible in the near future.

## 3 Cardioprotection by gasotransmitters

Two more widely studied gasses, namely nitric oxide (NO) and hydrogen sulfide (H_2_S), have demonstrated salutary effects in ischemic myocardium. NO is produced endogenously in a wide variety of mammalian tissues by nitric oxide synthase (NOS) as well as through non-enzymatic reactions. Its major biological actions include cyclic GMP-induced vasorelaxation and regulation of angiogenesis. Sublingual and parenteral formulations of NO donor compounds such as nitroglycerin have been used for over 100 years in the treatment and prevention of ischemic heart disease such as angina. NO has also been shown to be an important mediator of pre- and post-conditioning – strategies for reducing ischemia-reperfusion injury by brief periods of ischemia and reperfusion prior to ischemia or after reperfusion [39]. On the other hand, gaseous NO is highly reactive, and can be toxic at higher doses. Inhaled NO at > 80 ppm can lead to methemoglobinemia [40], and NO toxicity has also been correlated with antiproliferative effects and DNA strand breakage [41]. In myocardium, NO accumulation during ischemia may also contribute to worsening reperfusion injury [42]. Hu et al. showed that upregulation of iNOS increased myocardial levels of nitric oxide and its byproduct, peroxynitrite, resulting in increased apoptosis and infarct size after ischemia-reperfusion, and that this effect was reversed by administration of an iNOS Inhibitor [43]. Several other studies showed that administration of NOS inhibitors during ischemia decreased infarct size [44,45]. Additionally, NO can prevent calcium influx through cGMP-dependent inhibition of Ca^2+^ channels, which depresses myofilament and cardiac contractility [46]. Thus the role of NO in myocardial ischemic injury remains somewhat perplexing and controversial.

Hydrogen sulfide (H_2_S), like CO, has long been considered a toxic gas. Exposure to H_2_S may occur in the oil and gas industry, sewage and animal waste handling, and construction industries, and is associated with mucosal irritation, olfactory paralysis, sudden loss of consciousness, pulmonary edema, and death [47]. Inhalation of H_2_S at concentrations as low as 100–250 ppm for only a few minutes can result in incoordination, memory and motor dysfunction, and olfactory paralysis, while concentrations above 500 ppm may result in coma [48]. However, H_2_S and H_2_S donors have recently been shown to have cardioprotective effects as well. Zhang et al. demonstrated that exogenous H_2_S protected rat cardiomyoblasts against peroxide-induced apoptosis [49], while others have shown that H_2_S has vasodilatory [50], antioxidant [51], and anti-inflammatory [52] effects in cardiac tissue. Unfortunately, the flammability and toxicity of gaseous H_2_S, not to mention its foul odor, somewhat limits its use as a therapeutic agent, while inorganic H_2_S donors such as NaHS and Na2S are limited by rapid dissociation rates and autoxidation [53]. Investigations of natural H_2_S donors such as garlic and synthetic donors is ongoing, but less is known about these therapies when compared to CO-releasing agents.

Collectively, CO, NO, and H_2_S, while all potentially toxic in their native gaseous forms, have been shown to have salutary cardiovascular effects in controlled doses [[54], [55], [56]]. The body of literature supporting the therapeutic use of H_2_S lags behind the evidence supporting NO and CO, and CO may have more wide-ranging effects than NO due to its influence on the ubiquitous HO axis as well as the fact that NO is highly reactive and thus has limited bioavailability compared to CO which undergoes little to no metabolism. The remainder of this review will focus on traditional and innovative CO delivery methods and their suitability for potential clinical trials. It is important to note that these three gasotransmitters and the enzymes that generate them endogenously are intimately interrelated with clear crosstalk, e.g. CO binds to nitric oxide synthase. There is an enormous and exciting literature base on CO to which the reader is referred [[57], [58], [59], [60], [61]]. They are outside the scope of this review, which would not be able to do the topic justice.

## 4 Inhaled CO

The most intuitive method of CO administration is via inhalation, after which the gas rapidly diffuses across the alveolar-capillary membrane to bind to hemoglobin in the lung and subsequently cross into the systemic circulation where it can be readily quantified by measuring serum carboxyhemoglobin (COHb) concentrations. At COHb concentrations >10%, patients can develop adverse reactions such as headache, musculoskeletal pain, fatigue, somnolence, and nausea, while concentrations >50% may result in coma, cardiopulmonary failure, and death. However, several recent clinical trials have shown that controlled doses of inhaled CO can predictably and reliably achieve low, nontoxic COHb concentrations. Most of these studies have used repeated doses of CO over time in an effort to demonstrate beneficial effects. In a study of COPD patients, 100–125 ppm of CO administered via a nonrebreather mask for 70 min on 4 consecutive days achieved maximal COHb concentrations of 4.5% with no reported adverse effects. There were no significant differences in sputum neutrophils or lung function testing with treatment [62]. In another study, 100–200 ppm of CO were inhaled over 2 h by patients with idiopathic pulmonary fibrosis twice weekly for 12 weeks. This achieved maximal COHb of 3.82% in the treatment group (compared to 2.62% in the placebo group) with no differences in adverse events, physiologic measures, or serum matrix metalloproteinase 7 levels [63]. A study of healthy individuals treated participants with 100 ppm of inhaled CO for 1 h for 5 consecutive days, achieving maximum COHb concentration of 3.3% again with no adverse events. The CO treated patients had increased levels of antioxidant enzymes HO-1 and superoxide dismutase in skeletal muscle biopsies taken before and after treatment, as well as increased expression of mitochondrial fusion proteins mitofusin 1 and 2 and OPA-1, but no significant effect of CO on maximal oxygen uptake by VO2 testing [64]. One study used a 1-time dose of 500 ppm CO for 1 h, resulting in COHb concentration of 7%, and then injected patients with LPS to simulate endotoxemia. One out of 22 patients reported a mild headache. There was no effect of CO on the LPS-induced inflammatory markers, tachycardia, or hypertension [65]. Importantly, there have been no clinical trials investigating the effect of exogenous CO in cardiac disease to date.

Taken together, these results show that modest elevations of COHb can be consistently induced with CO inhalation, but that beneficial effects may be difficult to detect at these low concentrations. Safe, consistent delivery of CO is of the utmost importance if inhalational methods are to be used in clinical trials, and it is acknowledged that there may be a small margin between a dose that leads to measurable therapeutic effects and the lower limit of toxicity. Recently, self-contained delivery systems specifically designed for CO investigational trials have been developed [66]. Furthermore, a multi-institutional phase I trial treating intubated patients with ARDS with inhaled CO showed that the rise in COHb following CO exposure could be accurately predicted by using the Coburn-Forster-Kane equation at doses of 100 or 200 ppm (R^2^ = 0.9205; P < 0.0001), at least in intubated patients [67]. Delivery systems such as those with built in safeguards and more predictable and titratable drug levels may allow investigators to target a higher COHb concentration, closer to 10%, without crossing the line into toxicity. Nonetheless, currently the inhalation method of CO delivery suffers from lack of target organ selectivity and dosage control. In open systems, both titratability and a lack of scavenging of CO complicate therapy, especially in non-intubated patients who may exhale CO back into the atmosphere in variable amounts. Due to deep-seated societal conviction that CO is dangerous, this has made clinical trials more challenging. Inhaled gas raises many very relevant concerns related to feasibility of delivery, the need for cumbersome pressurized cylinders, and the unnecessary risk of exposure of healthcare workers. New modes of delivery have emerged including an oral CO delivery liquid [68], as gas enabled materials [69], CO Releasing Molecules or CORMs [61] as well as a new approach of organic prodrugs that allow for better tunability and tissue targeting [70]. The mechanisms of action of CO are diverse with reports of both heme and non-heme targets (Fig. 3).

## 5 Carbon monoxide Releasing Molecules (CORMs)

CORMs, pioneered by Motterlini, Mann and Foresti were developed in an effort to improve the safety and mode of delivery of CO delivery [61,71]. These are compounds capable of releasing controlled amounts of CO within the body. Most CORMs take advantage of the affinity of CO for metal compounds, and contain transition metals such as manganese (CORM-1), ruthenium (CORM-2, CORM-3), or molybdenum. These compounds have been shown to have cardioprotective effects in cell lines and small animal models of myocardial ischemia [[72], [73], [74]]. The most widely investigated compound is CORM-3, due to its solubility in aqueous solutions, but other water soluble compounds have been reported [75,76]. CORM-3 protected cardiomyocytes against cell injury caused by hypoxia-reoxygenation and paraquat, a reactive oxygen species generator that promotes oxidative stress [77]. CORM-3 also improved myocardial perfusion and contractility and reduced infarct size in an isolated rat heart model, effects which were abolished by 5-hydroxydecanoic acid, an inhibitor of mitochondrial ATP-dependent potassium channels [78]. The same authors showed that CORM-3 prolonged the survival time of transplanted mouse hearts when administered to the recipients for 8 days post-transplant. CORM-3, while unique in that it is soluble in aqueous solutions, suffers from wide variability in half-life depending on its solvent: its half-life in distilled water, phosphate buffered saline, and human plasma is 98 h, 20.4 min, and 3.6 min respectively [79]. Thus the release of CO using small molecule approaches, which also holds true for all forms of CO treatment regimens, remains difficult to predict and target to specific tissues. Clear differences in routes of delivery, carrier molecules and ultimately differences in pharmacokinetics and pharmacodynamics will have direct impact on the potential for CO toxicity and these aspects are currently constraining clinical applications. Other innovative agents have since been developed by Motterlini and Foresti known as HYCO molecules or hybrid CORM, which are designed to deliver CO and induce HO-1 [80].

Recently, CORMs have been designed to hold on to their CO payload until certain physicochemical conditions are met, potentially allowing them to be targeted to specific tissues. Several CORMs containing manganese, tungsten, or iron release CO in response to exposure to UV light, but these are limited either by insolubility in aqueous solutions or ligand toxicity, in addition to the obvious phototoxicity and limited penetration depth of UV irradiation [[81], [82], [83]]. Other molecules have been developed that release gases in response to magnetic field [84], ultrasound [85], or heat [86]. Still other CORMs release CO in response to internal stimuli present in target tissues (ie, tumors) such as peroxide [87] and glutathione [88]. Other CORMs are activated by enzymes found only in cell nuclei, potentially allowing for intracellular targeting of CO delivery [89]. These stimuli-responsive CORMs may improve CO targetability while decreasing systemic toxicity of the gas, allowing for delivery of higher concentrations of CO to desired tissues. This becomes very important when considering the heart because of the high amounts of myoglobin and the uniquely high mitochondrial metabolic rate and oxygen demand.

Despite these encouraging findings, a major barrier to using CORMs in clinical trials is the concern for toxicity of organometallic compounds in humans. Nearly all prior studies on CORMs have been carried out *in vitro*, in non-survival models, or short-term survival animal models. Though many studies utilizing CORMs have not reported significant cytotoxicity, there is some evidence that the accumulation of metal ligands after CO released can be cytotoxic. CORM-2, a ruthenium-containing CORM, was shown to induce significant cellular toxicity in the form of decreased cell viability, abnormal cell cytology, increased apoptosis and necrosis, cell cycle arrest, and reduced mitochondrial enzyme activity in rat cardiomyocytes, human embryonic kidney cells, and canine kidney cells, independent of the action of CO [90]. Other CORMs, even those containing non-heavy metals such as iron, demonstrate significant cytotoxicity in tumor cells, likely related to the effects of not only CO but the metallic ligand as well [83]. While desirable as a potential cancer therapeutic, this cytotoxicity could be harmful in normal tissues, limiting the use of certain CORMs in anti-ischemic or anti-inflammatory applications in myocardium. Importantly, recent reports have suggested that there are non-CO-related effects that can be attributed to many of these molecules due to their inherent reactivity characteristics [91].

## 6 Carbon monoxide releasing nanomaterials and delivery devices

Nanoparticle-based CORMs have been developed recently with specially engineered properties to enhance solubility, modulate the pharmacokinetics, reduce metal-compound toxicity, and more specifically deliver CO. Water-insoluble CORM-1 and CORM-2 have recently been embedded into nanopolymers to make them water-soluble, extending their half-lives in the process [92,93]. Another class of nanoparticles engineered with large pores or cavities increases the delivery of CORMs to the target disease site, while also reducing the potential toxicity related to metal compounds by trapping the metal fragments in their structure [90,92,94]. The poor targetability of traditional CORMs is due to random diffusion after administration, requiring large doses of CORMs to reach an effective CO concentration in target tissues. Several groups have engineered CORMs with optimized size and chemical properties to avoid rapid renal clearance or target specific tumor types [95,96]. Similarly, Qureshi et al. showed that CORM-2 loaded into lipid nanoparticles to increase the solubility and prolong the release of CO, leads to increased anti-inflammatory effects in a rat model of paw edema [97].

Studies regarding cardiovascular applications of nanomaterial CORMs are, as yet, quite limited. Gonzales et al. showed that MnCO bound to Al-MCM-41, a porous polymer nanomaterial, caused a vasodilatory effect on isolated mouse aorta rings, in a more sustained fashion than MnCO alone [98]. Another study using a rat photothrombotic model of cortical ischemia showed that administration of a Ruthenium-CO composite nanomaterial in conjunction with near-IR radiation induced local vascular dilation of >50%, and also decreased cortical infarct size [99]. Interestingly, Wollborn et al. demonstrated in a swine model of extracorporeal membrane oxygenation (ECMO) that a molybdenum-containing CORM successfully raised systemic COHb levels to a target of 10–13%, while using a silicone filter to prevent the heavy-metal byproducts from entering systemic circulation, suggesting that CORMs could be used safely in ECMO patients with the appropriately designed safeguards [100]. Swine on ECMO treated with CORM had less myocardial edema and troponin release after several hours on ECMO. These studies, while still pre-clinical, do demonstrate the potential for safe and effective CO targeting by nanoparticle-containing CORMs.

In elegant studies by Steiger et al., the delivery of CO was incorporated into an Oral Carbon Monoxide Release System designed for local delivery to the GI tract where a CORM could be incorporated into a miniaturized, orally delivered device [101]. This allows safe and effective delivery of CO without concern for carrier molecule effects. CO exposure using this approach protected against experimental colitis [101]. The utility of this method of delivery to treat pathology outside the intestine remains to be assessed, but in a modified approach a CORM was incorporated into the extracorporeal membrane oxygenator to deliver CO directly into the blood during cardiac arrest in a pig model. This again eliminated having to expose the animal to the entire molecule, and was effective at reducing acute kidney injury resulting from the cardiac arrest [102].

## 7 CO prodrugs

A relatively new approach to delivering CO as a pill are molecules pioneered by Wang et al. who have developed numerous organic compounds that release CO under physiological conditions and in response to various exogenous and endogenous triggers such as water, chemical reagents, esterase, ROS, and changes in pH. The compounds are metal free, highly tunable in terms of release rate and the ability to release CO with an endogenous (e.g. enzyme) or exogenous (e.g. chemical) trigger under physiological conditions. Additionally, they offer very unique opportunities to create compounds that can be targeted and enriched in specific organelles like mitochondria and can protect against acute liver failure [103] or acute kidney injury [104]. Prodrugs, similar to CORMs are amenable to multiple routes of administration and able to elevate systemic CO levels with an acceptable therapeutic index. Thus CO prodrugs offer promising pharmaceutical properties in terms of oral CO delivery and minimal drug accumulation in the body. One confounding challenge is the need to understand and characterize the non-CO portions of the molecules, which can be very complicated and interfere with development of the lead compounds should the metabolism prove to be complex from a pharmacologic and toxicologic standpoint [91]. Nevertheless, CO in pill form would be ideal in terms of physician and patient compliance. Thus there have been continued efforts to deliver CO reliably, safely and in a manner that is amenable to patient and physician.

## 8 Enteral CO

Hillhurst Biopharma has developed a drink comprised of a CO-saturated solution (HBI-002) in Generally Regarded As Safe (GRAS) ingredients. This allows for consumption of precise amounts of CO that poses no risk to healthcare workers, is stable, and carries no concerns of metabolic byproducts or off-target effects of carrier molecules. HBI-002 has been shown to be effective in preventing vasoocclusive crises associated with sickle cell disease and acute kidney injury [68,105]. HBI-002 has now been approved for use in FDA Phase I trials. The data showing inhibition in vaso-constriction and acute ischemic injury strongly suggests HBI-002 would be beneficial in myocardial infarction where vascular occlusion and tissue ischemia underlie the primary pathology. CO has been shown to promote tissue regeneration and thus is a viable treatment strategy in treating post ischemic insult [106]. In yet another development, researchers at MIT and BIDMC have created a CO-foam and a CO-encased sugar formulation, both of which are comprised of GRAS ingredients and can deliver CO across a much greater dose range due to taking advantage of the particular materials used. Termed Gas Entrapped Materials or GEMS, CO can be released under tight control across various epithelial barriers including the stomach and bowel [69].

## 9 Potential for future clinical trials

Each of the routes of administration discussed above have barriers to their potential use in clinical trials. The ideal CO delivery method for clinical use in myocardium would take into account: **1**) safety; achieving therapeutic concentrations of CO with no risk for accidental release into the atmosphere, potential CO overdose in the subject, adverse cardiac events or long term systemic toxicity from byproducts of CO delivery; **2**) targetability; specifically delivering CO to myocytes and/or cardiac vasculature; and **3**) temporal predictability; with rapid onset of delivery in conditions of acute myocardial ischemia, or prolonged, sustainable delivery in conditions of chronic ischemia where local delivery could be achieved without elevating levels systemically. None of the previously discussed methods of administration satisfies all three of these criteria, explaining the dearth of clinical trials of therapeutic CO to date.

Ischemic cardiac disease offers several unique opportunities for alternative CO delivery strategies for ischemic cardiac disease. For example, diagnostic and therapeutic interventions for acute coronary syndrome present the possibility of local CO delivery directly to ischemic tissue. Dissolved CO or a CORM could be delivered directly into reperfusing myocardium via a coronary catheter after circulation is restored via percutaneous coronary intervention or coronary stenting. Alternatively, a patient undergoing open-chest cardiac surgery could have CO delivered directly into the coronary arteries, or retrograde through a coronary sinus cannula, while the heart is arrested and the aorta is cross-clamped, protecting the rest of the body from the treatment. Open-chest surgery offers the additional possibility of administering topical preparations of CO, perhaps dissolved in a foam or hydrogel (e.g. GEMs), directly onto the myocardium, which have been successful in the skin and may be effective as a locally delivered formulation [107]. Finally, in chronically ischemic myocardium, osmotic pumps have been implanted between the rib cage and the heart to slowly administer drug into the ischemic territory over a period of several weeks [108]; one could envision loading such a pump with a stable CORM or CO liquid and allowing it to slowly diffuse directly into the at-risk myocardium.

## 10 Summary

Collectively CO has been studied for well over one hundred years, eighty of which defined CO as a poison. Since the early nineties, multiple laboratories have presented a compelling argument that CO is also powerfully cardioprotective. Akin to all drugs, there are ranges of therapeutic efficacy. Before we dismiss CO as only harmful, we must consider the data. Is CO hazardous because it was defined as a part of exposure to leaky furnaces or exhaust where multiple agents are in play that are cardiotoxic? Is the safety concerns only limited to those with compromised hearts? Why does the reported clinical safety data show no adverse events related to the heart and perhaps most importantly, why is CO protective in animal models of cardiac disease and injury? The answer likely lies somewhere in the middle, particularly when considering human pathophysiology. Careful Phase I safety data as well as early Phase II data suggests CO can be administered without adverse events [67,109]. Decades of research have been dedicated to the therapeutic use of carbon monoxide, and clinical trials are now ongoing. There are still hurdles to overcome, such as concerns related to appropriate dosing, and investigator safety, but the prospect of novel delivery modalities gives this gasotransmitter a legitimate opportunity as a therapeutic. With the development of prodrugs, drinks and foam delivery systems as well as further understanding of mechanisms of action, the next decade of CO science will undoubtedly be thrilling and prove that an idea mired in historic outdated literature can emerge and be critically reevaluated and perhaps lead to a change of heart.

## Declaration of competing interest

L. Otterbein is a scientific advisor to Hillhurst Biopharma.
